# Urological tumors treatment in Brazil during the SARS-Cov-2 outbrake

**DOI:** 10.1590/S1677-5538.IBJU.2020.0479.1

**Published:** 2021-02-03

**Authors:** Luciano A. Favorito

**Affiliations:** 1 Universidade do Estado de Rio de Janeiro - Uerj Unidade de Pesquisa Urogenital Rio de Janeiro RJ Brasil Unidade de Pesquisa Urogenital - Universidade do Estado de Rio de Janeiro - Uerj, Rio de Janeiro, RJ, Brasil; 2 Hospital Federal da Lagoa Serviço de Urologia Rio de Janeiro RJ Brasil Serviço de Urologia, Hospital Federal da Lagoa, Rio de Janeiro, RJ, Brasil

## COMMENT

SARS-CoV-2 pandemic has a great impact in all medical fields and the paper of Anjos Silva and Collegues from Brazil (1) shows interesting data about surgery in patients with urological tumors. Previous studies shows that SARS-CoV-2 changes the urologic practice in several fields (2-5), but the treatment of urological tumors during the outbreak has specially importance (6-8).

In this interesting paper the authors studied if uro-oncological surgeries at pandemic are safe in a cohort of 213 patients (161 patients were submitted to elective surgery and 44 to emergency surgery) in a reference hospital in Brazil and concluded that Elective uro-oncological procedures at the COVID-19 epidemic period in a COVID-19-free Institute are safe, and patients who need urgent procedures, with a long period of hospitalization, need special care to avoid COVID-19 infection and its outcomes.

We would like to congratulate the authors by the interesting and important report in this paper.
